# Ricotta cream: classification based on moisture and fat content considering general standards for cheeses and cream cheeses

**DOI:** 10.1016/j.heliyon.2021.e08408

**Published:** 2021-11-16

**Authors:** Mykaell Yan Muniz de Souza, Felipe Barbosa Cavalcanti, Elainy Virgínia dos Santos Pereira, Flávia Carolina Alonso Buriti, Eliane Rolim Florentino

**Affiliations:** Universidade Estadual da Paraíba, Núcleo de Pesquisa e Extensão em Alimentos, Campina Grande, Paraíba, Brazil

**Keywords:** Cream cheeses, Food standards, Adequacy to labelling of fat, High fat cheeses, Very high moisture cheeses

## Abstract

Ricotta cream though an emerging product sold in Brazil, by 2021 it has no fixed quality standards, a condition that can result in products with variable composition and properties. Additionally, there are no methods of sampling or analysis for its official control. In this context, this study investigated the physicochemical quality of five Brazilian ricotta cream brands to verify the extent of differences in the composition of this product, emphasizing the characterization and classification according to the Brazilian legislation and the Codex Alimentarius standards. Significant differences between brands concerning pH, titratable acidity, moisture, ash, fat, and fat in dry matter (FDM) were observed (P < 0.05), which were probably a result of their heterogeneous ingredient composition. According to Brazilian regulatory standards, all samples fit the “very high moisture” definition, and the brands A, B, D and E achieved the classification of “high-fat cheeses” since they contained at least 60.0% of FDM. Brand C was the only product that could be classified as a “medium fat cheese” due to having FDM values between 25.0% and 44.9%. All samples fit the Brazilian and Codex Alimentarius classification for “cream cheeses” based on their moisture, dry matter content, moisture on a fat-free basis and FDM. The results reinforce the need for regulatory standards regarding the physicochemical quality and composition of this cheese variety, to guarantee more transparency for the consumers and that they have access to more homogeneous products.

## 1 Introduction

“Ricotta cream” is a milk derivative product found in Brazil that has emerged over the last decade in the Brazilian market as a tasty option for consumers who enjoy dairy products (Gusso et al., 2012). This product is the result of the homogenization of ricotta cheese with additional ingredients, such as milk cream and polysaccharide gums, achieving a soft and spreadable texture (Fritzen-Freire et al., 2013).

According to data obtained in Brazil by the National Institute of Metrology, Standardization and Industrial Quality (INMETRO) for the fat composition in the whole matter of ricotta cheese (main constituent of ricotta cream), it was verified that the cheese has an average of 14.70 g/100 g total fat, 8.64 g/100 g saturated fat and 84.53 mg/100 g cholesterol (Instituto Nacional de Metrologia, Normalização e Qualidade Industrial, 2011). Due to its low fat and salt contents, high amount of protein and easy digestibility, ricotta could attend to the demands of consumers and the market for light and healthy products. Ricotta can be consumed as a soft cheese and also is frequently used as an ingredient in dishes and desserts (Sattin et al., 2016).

“White cheeses”, popularly considered as suitable for cardiovascular health, should be consumed carefully according to the Brazilian Society of Cardiology (SBC), as the preferential choice should be for cheeses low in saturated fat (Simão et al., 2013). This is because saturated fat should correspond to less than 10% of the daily energy intake, according to the World Health Organization (WHO), as part of a strategy for preventing chronic diseases (World Health Organization, 2003). Due to this fact, the SBC recommends paying attention to the fat content provided on food labels and to avoid the unrestricted intake of white cheeses popular in Brazil (Simão et al., 2013).

On the other hand, important benefits have been recently attributed to the milk fat associated to the dairy matrix, dairy calcium, milk fermentation, and/or probiotic microorganisms, such as lowering weight gain, inflammation, liver fat and the risk of central core obesity, and increasing hepatic and systemic insulin sensitivity (Mohan et al., 2020). Part of these benefits of milk fat might be attributed to the presence of fat-soluble vitamin D, medium chain saturated fatty acids and branched chain fatty acids (Bergamaschi and Bittante, 2017; Mohan et al., 2020).

Contrary to these recent findings, there is still a general recommendation from Brazilian health authorities and international dietary guidelines to avoid frequent consumption of fat cheeses and cream cheeses, such as ricotta cream. This is particularly due to their saturated fat and cholesterol content, since they are products of animal origin (Houston et al., 2008; Simão et al., 2013; US Department of Health and Human Services and US Department of Agriculture, 2015). This aspect was reinforced particularly after the Brazilian household sample survey (Instituto Brasileiro de Geografia e Estatística, 2011) that revealed an increase in the consumption of processed food, most of them higher in fat, sugar, and sodium, seen as “convenience foods”, despite their low nutritional quality, this in parallel to a decrease in the consumption of unprocessed and minimally processed foods, such as fruits, vegetables, cereals, and legumes. According to Block et al. (2017), the establishment of food standards and regulations, allied with adequate nutrition labelling, and the effective inspection of the products, are among the strategies to allow the population to have access to healthier food products for improvement of their dietary habits.

Most of the previous studies available in literature have focused their attention on nutritional composition, storage, microbiological activity and shelf life of the ricotta cheese (Borba et al., 2014; Sattin et al., 2016; Spanu et al., 2016, 2018) and a recent regulatory standard was approved in Brazil for this product (Brasil, 2020a, b). However, no fixed quality standards concerning composition, designation, hygiene requirements, packaging and labeling is available for ricotta cream yet and there are no methods of sampling or analysis for its official control. Therefore, this lack of quality standards results in products that vary in composition and properties, despite this some options of ricotta cream claimed as “light”, having 10% fat, are available in the Brazilian market. Moreover, it is difficult to establish standards for the regular ricotta cream version because there is still little research dealing with the characterization of this product (Gusso, 2013). Since ricotta cream is a ricotta cheese-based spreadable product, its sensory characteristics and, in some extent, its composition is closely related to cream cheese, an unripened acid-lactic coagulated cheese, with a soft texture together with mild-acid and creamy flavor (Chandan, 2011).

Knowing about the composition of ricotta cream is very important for defining the quality that this product offers to consumers and its safety to their health. A standardization of this product could make the food choice easier to help the consumer purchase foods with a balanced nutrient composition. Considering these aspects and the need of information to contribute to the conformity of ricotta cream, the aim of this study was to investigate the physicochemical properties and composition of different brands of ricotta cream commercialized in Brazilian markets, aiming to classify the samples concerning the identity and quality parameters for cheeses of Brazil and the Codex Alimentarius standards (Brasil, 1996, 2020a, b; Codex Alimentarius, 2010), as well as evaluate the adequacy of fat content to labeling according to the most recent Brazilian standards for food labeling (ANVISA, 2020) and those also adopted by the other members of the Southern Common Market (MERCOSUR) (Mercado Común del Sur, 2003).

## 2 Materials and methods

### 2.1 Collection of samples

Three different batches (genuine replicates) of five different brands of ricotta creams, denoted A, B, C, D and E, were purchased in grocery stores and supermarkets in the city of Campina Grande, Paraíba State, Brazil, from June to November 2014. The study was limited to these five brands because they were the only products with federal inspection available in the outlets at the time of the sample collection. All purchased samples were found in the outlets in refrigerated conditions, properly sealed, before the expiration date and in suitable condition for consumption. In most of the cases, the batches of the same brand were purchased in different supermarkets. For all cases, the samples within a same batch were randomly chosen, transported to the laboratory in their original plastic packages and kept sealed under refrigerated conditions (at 4 ± 1 °C) until the time of analysis.

### 2.2 Physicochemical analysis

The analysis of pH, titratable acidity, ash, fat, moisture, and dry matter were carried out for the three different batches of each brand of ricotta cream. The pH values were determined in triplicate for each batch with a pH meter (model *m*PA 210, MS Tecnopon, Piracicaba, Brazil) (Ardö and Polychroniadou, 1999). Titratable acidity was determined in triplicates for each batch, by mixing the ricotta cream with ethanol (95%), filtering, and titrating an aliquot of the filtrate with NaOH (0.1 mol/L) using phenolphthalein as the indicator, according to the appropriate standard methods and expressed as percent of lactic acid (AOAC International, 2019). Ash was determined gravimetrically by heating duplicate samples of each batch at 550 °C (AOAC International, 2019). Fat was determined, in triplicate samples for each batch, using a milk butyrometer after digesting the ricotta cream with H_2_SO_4_ (D = 1.5 g/ml) at 250 °C, the digested solution was mixed with isoamyl alcohol, and centrifugated the digested material in the butyrometer at 1200 rpm (Instituto Adolfo Lutz, 2008). Moisture and dry matter of samples were determined in triplicates for each batch using an automatic moisture analyzer, consisting of a semi-analytical balance with an infrared dryer (model ID 200, Marte Comércio de Instrumentação Analítica, São Paulo, Brazil). Fat in dry matter (FDM) was calculated, in triplicate, as the percent (g/100 g) ratio of the percentage of fat in the whole matter obtained for a sole batch of a brand (g/100 g) to the percentage of dry matter obtained for the overall mean of the same brand (g/100 g).

### 2.3 Classification of ricotta cream samples and adequacy of fat content for labeling

The data obtained for moisture and FDM of ricotta cream samples were compared with the Brazilian standards for cheeses (Brasil, 1996) and cream cheeses (Brasil, 2020a, b), as well as the Codex Alimentarius (2010) standards for cream cheeses, in this case, also using moisture in fat free basis (MFFB), calculated according to Eq. (1):

(1) $$\text{MFFB ​}=\text{ ​}\frac{\text{Moisture ​}\left(\text{g}/100\text{g}\right)}{100\text{ ​}-\text{ ​Fat ​}\left(\text{g}/100\text{g}\right)}\times 100$$

where the percent (g/100 g) of moisture is the moisture content obtained for the overall mean of a brand and the percent (g/100 g) of fat is the fat content in the whole matter obtained for the overall mean of the same brand.

The analytical data obtained for fat of samples were compared with the information described for this nutrient on the labeling of each ricotta cream brand to evaluate its adequacy according to recent Brazilian standards for food labeling (ANVISA, 2020) and those also adopted by the other members of the Southern Common Market (MERCOSUR) (Mercado Común del Sur, 2003).

### 2.4 Statistical analysis

The analytical results were expressed as means ± standard deviation. Differences between the batches for each brand and between the overall means of the five brands were statistically analyzed using analysis of variance (ANOVA), followed by the Tukey post-hoc test, using α = 0.05. Before the ANOVA evaluation, data was checked for the normality and homogeneity of variances, using the Shapiro-Wilk and Hartley tests. When this assumption was not verified, the equivalent non-parametric tests were applied, Kruskal-Wallis ANOVA followed by the Mann-Whitney U test, using α = 0.05.

## 3 Results and discussion

### 3.1 Label description and physicochemical analysis of ricotta creams

The information described in the labels of the evaluated ricotta cream brands concerning inspection type, origin, and ingredient composition is shown in Table 1. The brands analyzed were produced in four different Brazilian states – Minas Gerais (Brands A and B), Bahia (Brand C), Paraíba (Brand D), and São Paulo (Brands A, B, and E), and they can be sold in all Brazilian territory due to their federal inspection. Brands A and B could be produced in two different Brazilian states. All ricotta cream brands were processed with more than one different ingredient (whey and milk cream appear as the only common ingredients in the labels of all ricotta cream studied). Except for Brands C and E, the three batches of the same brand showed expiration dates of less than one-month intervals (data not shown), indicating these batches had been produced within a one-month period or less. Even within a short interval of production, the physicochemical analysis showed a heterogeneous composition between the batches for three of the five brands of ricotta cream studied. This will be described in greater detail later.

**Table 1:** Type of inspection, origin and ingredients of five commercial ricotta cream brands according to their labeling information.

Brand	A	B	C	D	E
Type of inspection	Federal	Federal	Federal	Federal	Federal
Origin (Brazilian state of manufacturing)	Minas Gerais and São Paulo	Minas Gerais and São Paulo	Bahia	Paraíba	São Paulo
Whey	+	+	+	+	+
Pasteurized skimmed milk	+	−	+	+	+
Whole milk powder	−	+	−	−	−
Milk cream	+	+	+	+	+
Lactic acid	+	+	+	+	−
Citric acid	+	−	+	−	+
Sodium chloride	+	−	+	−	+
Calcium chloride	−	−	−	−	+
Sodium bicarbonate	−	−	−	+	−
Guar gum	+	+	−	+	+
Xanthan gum	+	+	−	+	+
Carrageenan gum	−	−	+	−	−
Chlorophyll dye	−	+	+	−	−
Potassium sorbate	−	−	+	+	+
Nisin	−	+	−	−	−

+ = Present; − = Absent.

The results of pH and titratable acidity obtained for the three different batches of each brand of ricotta cream are shown in Table 2. Within the same brand, the mean values of pH did not differ significantly for the three batches evaluated (p > 0.05). The overall means of the pH values, considering all batches for each brand, ranged from 6.25 to 7.06, and there was no significant difference between Brands A, B, C, and E (p > 0.05). Brand D, in contrast, showed the highest pH values and differed significantly from the others (p < 0.05). In general, the pH of the ricotta creams studied was considered high and, combined with their high moisture values, as discussed later, this characteristic result in highly perishable products and makes them susceptible to microbial spoilage (Jafarzadeh et al., 2021). Except for Brand D, the results of pH observed in this study were close to those obtained by Gusso (2013) for ricotta creams developed with different thickeners, such as carrageenan gum, guar gum, tara gum, and xanthan gum (pH between 5.99 and 6.19), during the storage of ricotta creams processed with different levels of milk fat and cheese whey powder (pH from 5.17 to 6.91), and for commercial ricotta cheese (mean pH of 5.22). Borba et al. (2014) found pH values ranging from 6.77 to 6.91 during the storage of creamy ricotta, a ricotta cheese with a creamy texture resulting from the proportion and composition the milk-whey mixture used in its production and the moisture content, fatty acid profile and protein hydrolysis of the resulting cheese, which was prepared in the study with a mixture of milk and cheese whey from goats and cows. Fritzen-Freire et al. (2013) obtained pH values varying from 5.06 to 6.22 during the storage of ricotta creams processed with free or microencapsulated probiotic *Bifidobacterium* BB-12.

**Table 2:** Mean pH and titratable acidity of commercial ricotta creams.

Item	Batches	Brands				
		A	B	C	D	E
pH	1	6.49 ± 0.06^a^	6.41 ± 0.01^a^	6.21 ± 0.03^a^	7.10 ± 0.04^a^	6.64 ± 0.07^a^
	2	6.19 ± 0.00^a^	6.33 ± 0.02^a^	6.20 ± 0.01^a^	7.12 ± 0.01^a^	6.43 ± 0.01^a^
	3	6.29 ± 0.02^a^	6.40 ± 0.01^a^	6.33 ± 0.03^a^	6.96 ± 0.05^a^	6.31 ± 0.00^a^
	Overall mean ± SD	6.32 ± 0.13^A^	6.38 ± 0.07^A^	6.25 ± 0.07^A^	7.06 ± 0.08^B^	6.46 ± 0.15^A^
Acidity (g/100 g lactic acid)	1	0.5681 ± 0.01^a^	0.2654 ± 0.01^a^	0.6276 ± 0.01^a^	0.1373 ± 0.00^a^	0.2271 ± 0.00^a^
	2	0.6150 ± 0.00^a^	0.3167 ± 0.01^a^	0.5711 ± 0.01^a^	0.1023 ± 0.00^a^	0.2552 ± 0.01^a^
	3	0.5877 ± 0.00^a^	0.3063 ± 0.00^a^	0.6381 ± 0.02^a^	0.1577 ± 0.00^a^	0.2335 ± 0.01^a^
	Overall mean ± SD	0.5903 ± 0.02^C^	0.2961 ± 0.02^B^	0.6123 ± 0.03^C^	0.1324 ± 0.02^A^	0.2386 ± 0.01^B^

SD = standard deviation.

^a^ Data sharing a same lowercase superscript letters, within a column, did not differ significantly between batches for a same brand (p > 0.05).

^A,B,C^ Within a row, different superscript capital letters denote significant differences between the overall mean of different brands (p < 0.05).

Regarding titratable acidity, most values were inversely correlated with the pH of the brands. Sample D showed the lowest acidity and differed statistically (p < 0.05) from the other brands. The highest titratable acidity values were verified for Samples A and C, which did not differ significantly (p > 0.05) from each other but differed significantly (p < 0.05) from samples B and E. No significant difference (p > 0.05) was verified for acidity between the B and E samples. Moreover, no significant difference (p > 0.05) was verified for the acidity of the batches within each brand. The lower acidity values observed for Brand D were close to those found by Gusso (2013) for commercial ricotta cream (0.11 g/100 g lactic acid) and for experimental ricotta creams prepared with different thickeners (0.13 g/100 g lactic acid). The intermediate acidity values verified for Brands B and E were close to those found by Gusso (2013) for ricotta creams that added 10 g/100 g fat and 22.05 g/100 g cheese whey powder (0.26 g/100 g lactic acid). Furthermore, the results for second and third batches of Brand B were similar to those obtained by Borba et al. (2014) for creamy ricotta prepared with a mixture of milk and cheese whey from goats and cows (0.3 g/100 g lactic acid). On the contrary, the higher acidity values of Brands A and C were close to those observed by Fritzen-Freire et al. (2013) at the end of refrigerated storage (5 ± 1 °C) of probiotic ricotta creams (near 0.50 g/100 g lactic acid at 45 days and higher than 0.60 g/100 g lactic acid at 60 days). These differences in the acidity values of the brands evaluated in this study may be explained by the variations of ingredient compositions and their proportions used in the processing of ricotta creams. Taking into consideration the acidity results and the information shown in Table 1, the high acidity values of Brands A and C may be a consequence of the simultaneous addition of lactic and citric acids to their formulations, while other brands added only one type of acid.

The results obtained in this study for moisture, dry matter, ash, fat, and FDM are shown in Table 3. It was verified that the different brands analyzed showed a heterogeneous composition. Dissimilar values were also observed within the batches of the same brand, particularly in Samples A and E for most of the parameters analyzed.

**Table 3:** Mean composition of commercial ricotta creams.

Item	Batches	Brands				
		A	B	C	D	E
Moisture (g/100 g)	1	69.37 ± 0.29^b^	68.57 ± 0.25^a^	70.07 ± 0.86^a^	71.93 ± 1.10^a^	71.63 ± 0.47^a^
	2	67.13 ± 0.85^a^	68.87 ± 0.25^a^	68.67 ± 1.07^a^	74.47 ± 0.38^b^	74.03 ± 0.12^b^
	3	68.80 ± 0.61^ab^	67.03 ± 1.21^a^	69.13 ± 0.29^a^	73.73 ± 0.91^ab^	76.83 ± 0.31^c^
	Mean ± SD	68.43 ± 1.14^A^	68.16 ± 1.06^A^	69.29 ± 0.93^A^	73.38 ± 1.35^B^	74.17 ± 2.27^B^
Dry matter (g/100 g)	1	30.63 ± 0.29^a^	31.43 ± 0.25^a^	29.93 ± 0.86^a^	28.07 ± 1.10^b^	28.37 ± 0.47^c^
	2	32.87 ± 0.85^b^	31.13 ± 0.25^a^	31.33 ± 1.07^a^	25.53 ± 0.38^a^	25.97 ± 0.11^b^
	3	31.2 ± 0.61^ab^	32.97 ± 1.21^a^	30.87 ± 0.29^a^	26.27 ± 0.91^ab^	23.17 ± 0.30^a^
	Mean ± SD	31.57 ± 1.14^A^	31.84 ± 1.06^A^	30.71 ± 0.93^A^	26.62 ± 1.35^B^	25.84 ± 2.27^B^
Ash∗ (g/100 g)	1	1.4331 ± 0.04^a^	1.8139 ± 0.04^a^	1.3728 ± 0.01^a^	2.0817 ± 0.01^a^	1.7257 ± 0.02^a^
	2	1.4645 ± 0.01^a^	1.3379 ± 0.34^a^	1.9768 ± 0.01^a^	2.0368 ± 0.02^a^	1.9524 ± 0.01^a^
	3	1.7765 ± 0.15^a^	1.5901 ± 0.00^a^	2.6222 ± 0.01^a^	2.1017 ± 0.00^a^	1.9642 ± 0.06^a^
	Mean ± SD	1.5580 ± 0.18^A^	1.5806 ± 0.26^A^	1.9906 ± 0.56^C^	2.0734 ± 0.03^D^	1.8808 ± 0.12^B^
Fat∗ (g/100 g)	1	18.36 ± 0.58^b^	18.97 ± 0.53^a^	13.24 ± 0.57^a^	14.92 ± 0.00^a^	15.28 ± 0.00^a^
	2	19.91 ± 0.00^ab^	18.79 ± 0.00^a^	12.58 ± 0.54^a^	15.80 ± 0.00^a^	15.59 ± 0.97^ab^
	3	18.2 ± 0.56^a^	19.76 ± 0.00^a^	13.26 ± 0.58^a^	15.79 ± 0.00^a^	16.66 ± 0.00^b^
	Mean ± SD	18.82 ± 0.91^C^	19.17 ± 0.52^D^	13.03 ± 0.59^A^	15.50 ± 0.44^B^	15.84 ± 0.79^B^
FDM (g/100 g)	1	59.95 ± 1.88^a^	60.37 ± 1.69^a^	44.24 ± 1.91^a^	53.10 ± 0.00^a^	53.86 ± 0.00^a^
	2	60.57 ± 0.00^a^	60.36 ± 0.00^a^	40.16 ± 1.73^a^	61.88 ± 0.00^c^	60.02 ± 3.75^b^
	3	58.33 ± 0.56^a^	58.72 ± 0.00^a^	42.96 ± 1.87^a^	60.11 ± 0.00^b^	71.90 ± 0.00^c^
	Mean ± SD	59.62 ± 1.64^B^	59.82 ± 1.18^B^	42.45 ± 2.41^A^	58.42 ± 3.94^B^	61.93 ± 8.16^C^

SD = standard deviation.

^a,b,c^ Within a column, different superscript lowercase letters denote significant differences between batches of a same brand (p < 0.05).

^A,B,C^ Within a row, different superscript capital letters denote significant differences between the overall mean of different brands (p < 0.05).

FDM: Fat in dry matter.

∗ Values in the whole matter.

Brands A, B, and C showed the lowest moisture values and differed significantly (p < 0.05) from Brands D and E. There was no significant difference between the moisture of Brands A, B, and C or between D and E. The moisture results for these two latter brands were like those found by Gusso (2013) for commercial ricotta cream (73.61 g/100 g moisture) and for experimental ricotta cream processed with different thickeners (70.94 g/100 g–73.61 g/100 g moisture). On the contrary, the same author also found lower moisture values (57.46 g/100 g–66.45 g/100 g) for ricotta creams processed with different levels of milk fat and cheese whey powder (Gusso, 2013). Moreover, the moisture and dry matter values found in this study for Brands D and E were also close to the results found by Borba et al. (2014) for creamy ricotta prepared with a mixture of milk and cheese whey from goats and cows over a 14-day storage period (73.81 g/100 g–74.59 g/100 g moisture) and by Fritzen-Freire et al. (2013) during 60 days of storage of probiotic ricotta cream (25.41 g/100 g–28.04 g/100 g dry matter). In relation to the mean values of moisture and dry matter within the batches of a same brand, significant differences were observed for Samples A, D, and E (p < 0.05).

For the ash content, significant differences (p < 0.05) were verified between the overall mean values of the brands, except for A and B, which showed lower values. Higher levels of ash were observed for Brands C and D. Meanwhile, the batches within a same brand did not differ significantly in ash content (p > 0.05). The ash values in the whole matter obtained in this study were close to those found by Gusso (2013) for commercial ricotta creams (1.18 g/100 g ash) and for experimental ricotta creams adding different thickeners (2.07 g/100 g–2.24 g/100 g ash). Nonetheless, higher ash content (2.35 g/100 g–2.92 g/100 g) was also obtained by the same author for ricotta creams adding different proportions of milk fat and cheese whey powder (Gusso, 2013). According to this author, the ash in ricotta creams is mainly associated with potassium, sodium, calcium, and chlorides, which are the mineral constituents of cheese whey, added salts, such as sodium chloride and calcium chloride, and preservatives, such as sodium sorbate.

The overall mean values of fat content in the whole matter differed significantly among brands (p < 0.05), except between samples D and E. Concerning the mean values of fat in the whole matter within the batches of a same brand, significant differences were observed for Samples A and E (p < 0.05). Brand C showed the lowest fat content, while the highest amount was verified for Brand B. The results of fat in this study were like those found by Gusso (2013) for commercial ricotta creams and experimental ricotta creams prepared with thickeners (14.50 g/100 g–18.69 g/100 g fat).

In relation to FDM values, Brands C and E differed significantly (p < 0.05) from all other brands. Meanwhile, the batches within Brands D and E differed significantly for this parameter (p > 0.05). Brand C showed the lowest FDM content, while the highest values were found in Brand E. Gusso (2013) obtained 62.96 g/100 g FDM, close to most of the values observed in the current study for ricotta creams processed without milk cream and cheese whey powder addition, while the FDM ranged from 29.18 g/100 g–50.85 g/100 g in a study for the formulations that added those ingredients.

The possible factors that might have contributed to the heterogeneous composition of ricotta cream are the differences in dairy herds, feeding system, weather changes, production region, processing, and kind and proportions of ingredients (Ozrenk and Inci, 2008; Bergamaschi and Bittante, 2017; Aljerf et al., 2018). These components could explain the differences obtained between the brands. According to Ozrenk and Inci (2008), there is a negative correlation between environmental temperature and the amount of milk fat since the solid fat tends to decrease when the temperature increase. Additionally, the season of production and its relationship with the composition of milk could explain the differences obtained between the different batches of a same brand. The seasonality of milk directly influences the total amount and composition of protein, fat and other nutrients (Bertocchi et al., 2014; Aljerf et al., 2018). The secretion of prolactin in the plasma is higher in the summer than the winter, which could be related to the reduction of the milk fat content in the summer, when there is a high light-to-dark ratio (Ozrenk and Inci, 2008).

### 3.2 Classification of ricotta cream samples and adequacy of fat content for labeling

The classification of ricotta cream brands based on the Brazilian legislation for cheeses and the standards of Codex Alimentarius for cream cheeses is summarized in Table 4.

**Table 4:** Summary of the classification achieved by commercial ricotta creams according to the standards consulted.

Brand	Standards					
	Brasil (1996)				Brasil (2020a, b)	Codex Alimentarius (2010)
	Very high moisture cheese (moisture content above or equal to 55.0 g/100 g)	Medium fat (25.0 g/100 g–44.9 g/100 g FDM∗)	Full fat (45.0 g/100 g–59.9 g/100 g FDM)	High fat or double cream (FDM content above or equal to 60.0 g/100 g)	Cream cheese (minimum content of 25 g/100 g FDM and maximum 78 g/100 g moisture)	Cream cheese (minimum content of 25 g/100 g FDM, 67 g/100 g MFFB∗∗ and 22 g/100 g dry matter)
A	Yes	No	Batches 1 and 3 only	Yes for batch 2	Yes	Yes
B	Yes	No	Batch 3 only	Yes for batches 1 and 2	Yes	Yes
C	Yes	Yes	No	No	Yes	Yes
D	Yes	No	Batch 1 only	Yes for batches 2 and 3	Yes	Yes
E	Yes	No	Batch 1 only	Yes for batches 2 and 3	Yes	Yes

∗ FDM: Fat in dry matter.

∗∗ MFFB: Moisture in fat free basis.

According to the Brazilian regulatory standards (Brasil, 1996), all brands of ricotta creams studied fulfilled the requirements for “very high moisture” cheeses, since they all measured higher than 55 g/100 g. Based on the overall means of FDM content and the Brazilian legislation (Brasil, 1996), Brands A, B and D should be classified as “full fat” cheeses, since they showed FDM between 45.0 g/100 g and 59.9 g/100 g. Nonetheless, considering the mean values of each batch, one batch of Brand A and two batches of Brands B and D did not fit in this requirement and should be classified as “high fat” or “double cream” cheeses due to an FDM content of 60.0 g/100 g or higher (Brasil, 1996). The FDM overall mean would allow Brand E to be classified as a “high fat” cheese, although one batch of this brand (Batch 1) showed FDM values that could allow it to be classified as “full fat” cheese (Brasil, 1996). Only Brand C fit in the requirements of Brazilian legislation for “medium fat” cheese due to the mean FDM values between 25 and 44.9 g/100 g (Brasil, 1996) for the three batches. The high FDM values observed for ricotta cream samples are probably a result of added milk cream, as according to Table 1, this ingredient was present in the formulations of all evaluated brands.

According to the standards for cream cheeses, the recent Brazilian standards (Brasil, 2020a, b) and the standards of Codex Alimentarius (2010), all samples analyzed in the current study could receive the classification for this category of product, due to a moisture content lower than 78 g/100 g according to the Brazilian standards, dry matter content higher than 22 g/100 g and the MFFB higher than 67 g/100 g according to the Codex Alimentarius, and also FDM higher than 25 g/100 g according to both standards. In this study, Brand C showed the lowest MFFB content (79.67 g/100 g) when compared to other brands evaluated (data not shown). It is important to reemphasize that lack of homogeneity of fat content among the brands and batches evaluated allowed them to be classified in more than one cheese category, including batches of the same brand.

Since there is a large concern for information about the fat content presented in labeling, particularly due to the main contribution of the nutrients to the total energy of foods, this study evaluated the adequacy of the analytical data of fat to those found in cheese labels using the regulatory standards adopted by the state members of MERCOSUR (Mercado Común del Sur, 2003) and those recently approved by the Brazilian legislation (ANVISA, 2020). With this purpose, the total fat content per serving portion (30 g), data given in the labeling of ricotta cream brands, their conversion to a 100 g serving portion, and their variation from the analytical data are shown in Table 5. Based on information shown in the labeling, Brand A would contain the highest fat content, while the lower fat values would be found in Brands C, D and E, which were not observed for the analytical data, except for Brand C. According to the MERCOSUR standards (Mercado Común del Sur, 2003), a variation of ±20% of the analytical data in relation to the nutrient content described in the labeling is acceptable. According to Brazilian standards (ANVISA, 2020) the variation of the products’ fat content cannot exceed 20% in relation to that mentioned in the labelling. Therefore, one batch of Brand E did not fill these requirements. Although most of the samples evaluated showed labeling information regarding fat in agreement with the regulatory standards consulted, borderline values (close to the allowed limit) were verified for two batches of Brand D and for the overall mean of Brand E. Brands A and C showed that the fat values declared in the labeling were more adjusted to the analytical data compared with the other ricotta creams.

**Table 5:** Total fat content in serving portions of 30 g and 100 g according to the information described in the labeling of commercial ricotta creams and their comparison with the analytical data.

Item evaluated	Condition	Brands				
		A	B	C	D	E
Total fat according to the labeling	g per 30 g serving portion	5.3	5.2	4.0	4.0	4.0
	g per 100 g serving portion	17.67	17.33	13.33	13.33	13.33
Difference of fat analytical data in relation to the fat content described in the labeling (%)	For batch 1 only	3.90	9.46	-0.68	11.93	14.63
	For batch 2 only	12.68	8.42	-5.63	18.53	16.95
	For batch 3 only	3.00	14.02	-0.53	18.45	24.98
	For the overall mean of three batches	6.51	10.62	-2.25	16.28	18.83

Due to the very high moisture, that turns the ricotta cream highly perishable, and the high fat content, that turns the product susceptible to lipid oxidation, it is important to reinforce the importance of enhancing the conditions of processing to avoid microbiological contamination, using proper packaging to preserve the product during its shelf life, including the protection from light and O_2_, enhancing the handling and marketing, as well as controlling the storage temperature to prevent the fast spoilage (Jafarzadeh et al., 2021).

## 4 Conclusion

This study showed the heterogeneous physicochemical composition between brands and batches of ricotta cream sold in Brazil. This lack of uniformity was probably due to the differences in the region of production, processing, and the kind and proportions of ingredients among other factors. This heterogeneity is also impacted by the absence of official quality standards for ricotta cream in Brazil. According to the Brazilian regulatory standards for cheeses, the products evaluated were classified in more than one category regarding fat composition, including batches of a same brand. Based on Codex Alimentarius standards, all ricotta cream samples evaluated were classified as cream cheeses. A variation higher than 20% of the analytical fat content data in relation to the fat content described in the labeling was verified for one batch of ricotta cream, which was not in agreement with the Brazilian legislation and MERCOSUR standards. Borderline values (close to 20%) were verified for two brands. This heterogeneity for the fat content is concerning since it can result in wrong choices by the consumers. The findings of the present study, therefore, reinforce the importance of regulatory standards of ricotta cream to guarantee more transparency to the consumers and their access to more homogeneous products. Despite the limited number of brands studied, the small number of batches sampled for each brand and the lack of evaluation of other characteristics of the ricotta cream, such as protein content, microbiological parameters, rheology, and sensory features, considered as the weakness of this study, the parameters here reported could be used as a basis to prepare the physicochemical and composition standards to be adopted for this product, which is considered the strength of this study.

## Declarations

### Author contribution statement

Mykaell Yan Muniz de Souza: Conceived and designed the experiments; Performed the experiments; Analyzed and interpreted the data; Wrote the paper.

Felipe Barbosa Cavalcanti: Performed the experiments.

Elainy Virgínia dos Santos Pereira: Performed the experiments; Analyzed and interpreted the data.

Flávia Carolina Alonso Buriti: Analyzed and interpreted the data; Contributed reagents, materials, analysis tools or data; Wrote the paper.

Eliane Rolim Florentino: Conceived and designed the experiments; Contributed reagents, materials, analysis tools or data; Wrote the paper.

### Funding statement

This work was supported by the 10.13039/501100007184Universidade Estadual da Paraíba (UEPB, grant 001/2021), 10.13039/501100003593Conselho Nacional de Desenvolvimento Científico e Tecnológico (CNPq, Project 477799/2012-4), 10.13039/501100002322Coordenação de Aperfeiçoamento de Pessoal de Nível Superior (CAPES), 10.13039/501100005669Fundação de Apoio à Pesquisa do Estado da Paraíba (FAPESQ, Project 028/2018), Fundação Parque Tecnológico da Paraíba (PaqTcPB) and Programa de Apoio à Pós-Graduação e Pesquisa of UEPB (PROPESQ/Call 2015).

### Data availability statement

Data will be made available on request.

### Declaration of interests statement

The authors declare no conflict of interest.

### Additional information

No additional information is available for this paper.
